# Targeted inhibition of *mecA* and *agrA* genes in clinical MRSA isolates by natural bioactive compounds

**DOI:** 10.3389/fmicb.2025.1643774

**Published:** 2025-08-26

**Authors:** V. Vinodhini, M. Kavitha

**Affiliations:** School of Bio Sciences and Technology, Vellore Institute of Technology, Vellore, Tamil Nadu, India

**Keywords:** antibiotic resistance, bioactive compounds, methicillin-resistant *Staphylococcus aureus*, quorum sensing, *mecA* gene, *agrA* gene

## Abstract

**Introduction:**

Methicillin-resistant *Staphylococcus aureus* (MRSA) colonization in the nasal nares significantly contributes to hospital-acquired infections. This poses a global health concern due to its resistance to β-lactam antibiotics and its virulence mediated by quorum sensing (QS). This study investigates the antimicrobial efficacy of two natural bioactive compounds (BACs)—curcumin and eugenol—against clinical MRSA isolates, specifically focusing on their impact on *mecA* and *agrA* gene expression.

**Methods:**

Nasal swab samples from 150 patients yielded 53 staphylococcal isolates, among which 25 were identified as MRSA. Three highly resistant isolates (VITKV25, VITKV32, and VITKV39), identified as *S. aureus* through 16S rRNA gene sequencing, were selected for further analysis. The antibacterial efficacy of curcumin, eugenol, and gallic acid was assessed through agar well diffusion and minimum inhibitory concentration (MIC) assays. The presence of *mecA* and *agrA* genes was detected using polymerase chain reaction (PCR). Additionally, the impact of these compounds on *mecA* and *agrA* gene expression was assessed using Quantitative real-time PCR (qRT-PCR).

**Results and discussion:**

The isolates exhibited resistance to multiple antibiotics, including ampicillin, oxacillin, vancomycin, cefoxitin, and erythromycin. Curcumin and eugenol distinctly downregulated *mecA* and *agrA* expression levels. In particular, curcumin completely suppressed *mecA* gene expression in VITKV39 (0.0) and *agrA* expression in VITKV25 and MRSA (ATCC 43300) (0.0 and 0.1, respectively). Eugenol reduced both genes, *mecA* and *agrA*, in VITKV32 to 0.01 and 0.02, respectively. These findings highlight the therapeutic potential of curcumin and eugenol as adjuncts to conventional antibiotics by targeting both resistance and virulence pathways in MRSA.

## Graphical abstract

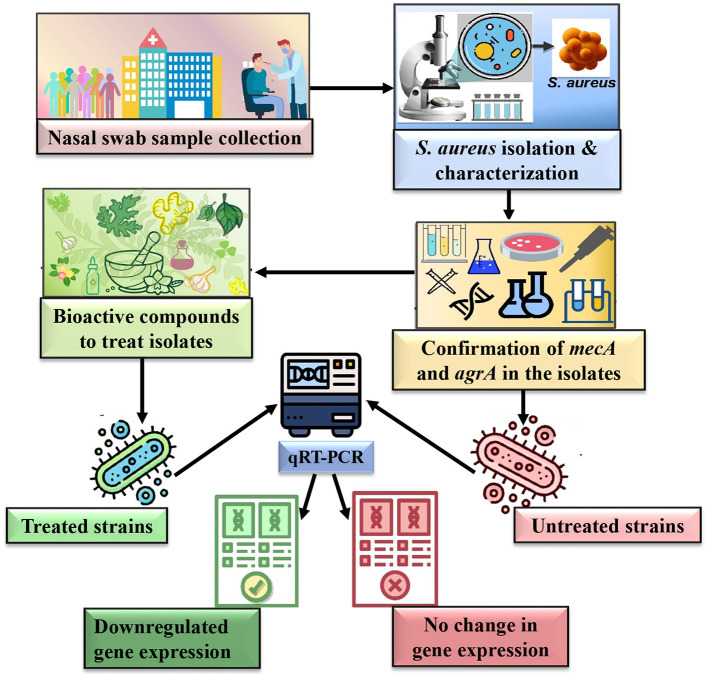

## 1 Introduction

*Staphylococci* are opportunistic pathogens that can cause infections in specific conditions, such as when the host's immune system is compromised or the skin or mucosal barriers are breached (Jaradat et al., 2020). Although many strains of *Staphylococci* are harmless and also part of the human body's normal flora, some strains may cause various infections, including skin diseases (boils, impetigo, and cellulitis), respiratory diseases, or indeed serious conditions like septicaemia, endocarditis, and pneumonia (Al-Dmour et al., 2023). The virulence arsenal and increasing multidrug resistance have been responsible for its adaptability, persisting in hospital and community settings. The nasal cavity is a critical site of both asymptomatic carriage and subsequent transmission of the organism, particularly in hospitals. Studies revealed that the nasal carriage rate can be as high as 60–70% among patients in hospitals and health care workers, significantly indicating the dynamic aspects of nosocomial infections (Rongpharpi et al., 2013). The *S. aureus* was the first organism to develop resistance to penicillin shortly after its introduction (Lowy, 2003).

The semi-synthetic β-lactam antibiotic, methicillin, was developed in 1959 as an alternative to penicillin, but MRSA was reported within 2 years. The emergence and widespread occurrence of MRSA strains have impelled the global antimicrobial resistance crisis. The *mecA* gene, which encodes penicillin-binding protein 2a (PBP2a), decreases the binding efficacy of β-lactam antibiotics to the target, leading to resistance in MRSA organisms. The *mecA* gene is located on the staphylococcal cassette chromosome mec (SCCmec), which contains distinct resistance determinants (Deurenberg et al., 2007; Urushibara et al., 2020). These features contribute to MRSA's multidrug-resistant phenotype and limit the number of therapeutic and management approaches for MRSA infections (Idrees et al., 2023). In addition to antibiotic resistance, *S. aureus* pathogenicity is strongly regulated by QS systems, particularly the accessory gene regulator (agr) locus, which governs key virulence factors such as adhesins, exotoxins, and degradative enzymes (Fuqua et al., 1994; Vinodhini and Kavitha, 2024). This operon is organized around two promoters, P2 and P3, that produce two transcripts, RNAII and RNAIII, respectively. The RNAII encodes AgrB, AgrD, AgrC, and AgrA—the core elements for autoinducing peptide (AIP) production and signal transduction. AgrC-AgrA represents a typical two-component system, where AgrC phosphorylates AgrA in response to AIP binding, thereby stimulating the transcription of RNAIII and virulence gene expression (Thoendel et al., 2011). Downregulation of AgrA disrupts this cascade, significantly reducing pathogenicity. Furthermore, numerous *in vivo* and *in vitro* studies have demonstrated that the inhibition of AgrA drastically attenuated virulence factors. Therefore, it may be possible to inhibit or reduce virulence by targeting AgrA, which would increase the pathogen's susceptibility to antibiotics. Additionally, the QS inhibitor would gain great significance if it receives FDA approval, as it might be a convenient adjunct to current antibiotics (Palaniappan et al., 2021). Consequently, targeting both *mecA* and *agrA* provides a promising strategy to simultaneously suppress resistance and virulence.

Bioactive compounds (BACs) of biological origin are known for their medicinal properties and role in preventing infectious diseases. The BACs play a key role in the production of novel synthetic drugs, ranging from anticancer treatments to antibiotics. They offer several advantages over the synthetic compounds, such as fewer side effects, a broader spectrum of action, lower cost, and abundant natural availability (Stan et al., 2021; Nandhini et al., 2023). BACs are mostly certain secondary metabolites, exhibit antimicrobial, immunomodulating, inflammatory, and antioxidant properties (Sytar and Smetanska, 2022). Secondary metabolites possessing remarkable activity against MRSA have been taken into consideration and investigated for their inhibitory activity. BACs have been selected in this study according to recent research outcomes and potential effectiveness against MRSA. All five compounds are secondary metabolite classes, including polyphenols, alkaloids, and terpenes. Curcumin is a polyphenolic compound derived from the rhizome of *Curcuma longa* L. and has been known to possess a wide range of medicinal properties, including antimicrobial activity (Batista de Andrade Neto et al., 2021). Gallic acid, a phenolic compound present in the bark, leaves, and roots of berries, nuts, and tea leaves, exhibits a wide range of biological activities, such as antibacterial, anti-inflammatory, anti-cancer, and antioxidant properties (Jiamboonsri et al., 2023). Piperine, a naturally occurring alkaloid in black pepper (*Piper nigrum*) has antioxidant, antiproliferative, antibacterial and immunomodulatory effects. Its antibiofilm effect against MRSA has been studied, highlighting its potential for developing novel antibiofilm therapies (Das et al., 2024). Eugenol (4-ally-2-methoxyphenol), a key component of clove oil, is widely used as a flavoring agent in culinary and cosmetic l. Studies have shown its antibacterial, antioxidant, anti-inflammatory, and antispasmodic properties (El-Far et al., 2021). α-Terpineol is a monocyclic terpene alcohol widely used in the pharmaceutical industry, is extracted from the essence of *Citrus aurantium* (neroli), *Melaleuca alternifolia*. Apart from possessing a well-established bactericidal action against both Gram-positive and Gram-negative bacteria, α-terpineol has analgesic, anti-inflammatory, antioxidant, and antiproliferative properties. And incredibly, it is effective against drug-resistant bacteria (Johansen et al., 2022).

To our knowledge, no studies have evaluated the effectiveness of the aforementioned BACs on the transcription levels of the *mecA* and *agrA* genes in MRSA isolates. In this context, the present study aimed to investigate the antimicrobial activity of curcumin, eugenol, and gallic acid against multidrug-resistant clinical MRSA isolates. Particularly, this study focused on their inhibitory effect on *mecA* and *agrA* gene expression by qRT-PCR. By examining phenotypic as well as molecular endpoints, this study affords intuitive insights into the potential of these natural compounds as supplemental therapeutics against resistant and virulent strains of *S. aureus*.

## 2 Materials and methods

### 2.1 Study design and sample collection

A cross-sectional comparative study was performed to determine prevalence rates of nasal carriage of *S. aureus*, especially methicillin-resistant strains. Totally 150 nasal swab samples were collected from patients at Sri Bala Nursing Home, Chennai, Tamil Nadu, India, irrespective of age, sex, or occupation. Samples were collected with sterile cotton swabs and placed into the anterior nares, where the swabs were gently rotated twice. The swabs were placed in sterile transport containers and processed within 6 h under aseptic conditions. Ethical clearance for this study has been obtained from the Institutional Ethical Committee for Human Studies, Vellore Institute of Technology, Vellore, India (Approval No. VIT/IECH/XI/2021/11). All participants gave written informed consent, and confidentiality was maintained throughout the study.

### 2.2 Isolation and preliminary identification of staphylococcal isolates

All nasal swab samples were streaked onto mannitol salt agar (MSA; HiMedia, India) and incubated at 37 °C for 24–48 h. Plates with yellow-colored colonies indicating mannitol fermentation were selected as presumptive staphylococcal isolates. Distinct colonies were sub cultured and subjected to preliminary identification through standard microbiological and biochemical methods. The cell morphology was determined by Gram staining. Catalase activity was tested with 3% hydrogen peroxide, while coagulase production was assessed with the tube coagulase test using rabbit plasma (HiMedia). Oxidase activity was determined using oxidase reagent discs (HiMedia). Deoxyribose production was evaluated in DNase medium (10g—Pancreatic digest of casein, 10g—yeast extract, 2g—deoxyribonucleic acid, 5g—NaCl, 15g—agar, pH 7.5), which was then flooded with 1N HCl solution to visualize the zone of clearance (Kateete et al., 2010). Isolates showing Gram-positive cocci in clusters, catalase-positive, coagulase-positive, oxidase-negative, and DNase-positive profiles were presumptively considered as *S. aureus*. Final confirmation was performed by 16S rRNA gene sequencing, as described in a subsequent section.

### 2.3 Antibiotic susceptibility test

The antibiotic susceptibility of the Staphylococcal isolates was determined by following the Kirby-Bauer disc diffusion method on Mueller-Hinton agar (MHA; HiMedia, India), by following Clinical and Laboratory Standards Institute (CLSI) guidelines (Beargie et al., 1965; Clinical and Laboratory Standards Institute, 2018). The isolates were inoculated in Luria-Bertani broth (HiMedia, India) and incubated at 37 °C overnight, the density of which matched the turbidity of 0.5 McFarland standard (~1.5 × 10^6^ CFU/mL). The bacterial suspension was uniformly swabbed onto the surface of MHA plates. Octo discs (HiMedia, India) containing ampicillin (10 μg), levofloxacin (5 μg), vancomycin (30 μg), gentamicin (10 μg), oxacillin (1 μg), clindamycin (2 μg), erythromycin (15 μg) was carefully placed at the center of the plates. The diameters of inhibition zones were determined after 24 h of incubation at 37 °C and interpreted using the CLSI-recommended breakpoints to categorize isolates as susceptible, intermediate, or resistant. For methicillin resistance screening, additional discs of methicillin (5 μg) and cefoxitin (30 μg) were used under the same conditions. Isolates showing resistance to either disc were considered presumptive MRSA and were further confirmed by a molecular approach. *S. aureus* ATCC 43300 and ATCC 25923 were used as MRSA and MSSA quality control strains, respectively, in all the experiments.

### 2.4 Molecular confirmation by 16S rRNA gene sequencing

For species-level confirmation, three staphylococcal isolates labeled as VITKV25, VITKV32, and VITKV39, exhibiting the maximum antibiotic resistance profiles, were selected. The strains were subjected to 16S rRNA gene sequencing using agarose gel electrophoresis and PCR analysis by the Rajiv Gandhi Centre of Biotechnology (RGCB), Kerala. The primers used for this process were 16S-RS-F 5′CAGGCCTAACACATGCAAGTC3′ as forward primer and 16S-RS-R 5′GGGCGGWGTGTACAAGGC3′ as reverse primer. The resulting sequences were compared with known bacterial taxa using the NCBI BLASTn tool. Phylogenetic relationships were further evaluated using MEGA11 software using the neighbor-joining method (Sada et al., 2024).

### 2.5 Biofilm formation assay

The biofilm-forming capacity of confirmed *S. aureus* isolates (VITKV25, VITKV32, and VITKV39) was evaluated using the crystal violet staining method in a 96-well microtiter plate. Each isolate was streaked onto trypticase soy agar (TSA; HiMedia, India) and kept at 37 °C for overnight incubation. A single colony from the TSA plate was inoculated onto 5 mL of trypticase soy broth (TSB; HiMedia, India) and incubated overnight at 37 °C. The overnight cultures were diluted 1:100 in fresh TSB, and 200 μL of the diluted suspension (adjusted to an OD_600_ of 0.5) was dispensed into a sterile, flat-bottom 96-well polystyrene microtiter plate (HiMedia, India). The plates were then incubated under static conditions at 37 °C for 16 h. After incubation, wells were washed three times with sterile distilled water to remove non-adherent cells, and plates were allowed to dry in blotting paper. Then, 200 μL of 0.1% crystal violet solution was added to the wells and incubated at room temperature for 30 min. The wells were blotted dry and washed with distilled water, and air-dried. To quantify the retained stain, 200 μL of 95% ethanol was added to each well to solubilize the bound dye, and spectrophotometric absorbance was taken at 540 nm using a microplate reader (Bio-Rad, USA). The results were an average of the three experiments (Shukla and Rao, 2013; Turk et al., 2013). Biofilm formation was categorized according to the mean OD values: OD <0.120 as non-biofilm producers, 0.120 – 0.240 as moderate biofilm producers, and >0.240 as strong biofilm producers (Kala et al., 2012). *S. aureus* ATCC 43300 (MRSA reference strain) and ATCC 25923 (MSSA reference strain) were included as strong and moderate biofilm-producing positive controls, respectively.

### 2.6 Agar well diffusion assay and MIC

The antibacterial activity of selected BACs—curcumin, piperine, eugenol, gallic acid and α-terpineol—was evaluated against VITKV25, VITKV32, and VITKV39 using agar well diffusion and minimum inhibitory concentration (MIC) assay. The selected bacterial strains were grown on Nutrient broth and the turbidity of the bacterial suspension was adjusted to 0.5 MacFarland's standard. The MHA plates were spread with 100 μl of the bacterial suspension. Using a sterile borer, 6 mm diameter wells were made in the inoculated agar. BACs, curcumin, piperine (HiMedia, India), eugenol, gallic acid and α-terpineol (Sigma-Aldrich, USA) were used to prepare stock solutions at a concentration of 100 mg/mL. The 50μl of each stock solution was loaded in the respective wells and the same volume of solvent (5% DMSO) was used as a negative control. The plates were incubated at 37 °C for 24 h and the zone of inhibition in diameter were measured in mm (Hossain et al., 2022).

The MIC of *S. aureus* was determined for three compounds—curcumin, eugenol and gallic acid which showed activity against the isolates, using micro-broth dilution method in 96 well plate following CLSI guidelines. In the 96 well plate, the compounds were serially diluted to obtain final concentrations of 10, 5, 2.5, 1.25, 0.62, and 0.31 mg/mL. Then, 90 μL of double strength MHB was transferred to each well, followed be the addition of 100 μL of the respective compound from each dilution. Finally, 10 μL of the respective bacterial culture (adjusted to 0.5 McFarland) was added to each well, and the microtiter plate was incubated at 37 °C for 24 h and bacterial growth was assessed by measuring absorbance at 600 nm using a microplate reader (Bio-Rad, USA). The MIC was defined as the lowest concentration of the compound that completely inhibited visible growth (Moses et al., 2020). MRSA and MSSA reference strains were used (ATCC 43300 & ATCC 25923) for both assays.

### 2.7 DNA extraction and detection of *mecA* and *agrA* genes by PCR

Genomic DNA was extracted from the isolates VITKV25, VITKV32, and VITKV39 using the phenol-chloroform method. Overnight cultures were grown in Luria-Bertani (LB) broth at 37 °C and centrifuged at 10,000 rpm for 5 min to pellet the cells. The pellet was resuspended in TE buffer (10 mM Tris-HCl, 1 mM EDTA, pH 8.0), followed by cell lysis using lysozyme (10 mg/mL) and proteinase K (20 mg/mL). after incubation at 37 °C for 1 h, DNA was extracted with an equal volume of phenol: chloroform: isoamyl alcohol (25:24:1) and precipitated using cold isopropanol. The resulting DNA pellet was washed with 70% ethanol, air-dried, and resuspended in nuclease-free water. DNA quality and concentration were assessed using 1% agarose gel electrophoresis and spectrophotometric quantification (Nanodrop, Thermo Fisher Scientific, USA) (Divyakolu et al., 2021).

The extracted DNA was used as a template DNA in PCR to detect the presence of the *mecA* and *agrA* genes. The effective primer pairs were designed from the FASTA sequences retrieved from the NCBI database, thereby validating their melting temperature, GC content, and other required specifications. The primer sequences (Eurofins, Bangalore) used in this study have been included in Table 1. The reaction mixture (10 μl) contained 2 μL template DNA, 1 μL forward primer (FP), 1 μL reverse primer (RP), 2 μL of Taq DNA polymerase master mix (Ampliqon, Denmark), and 4 μl of nuclease-free water for both genes. Amplification of *mecA* gene were performed for 30 cycles under the following cyclic conditions: initial denaturation at 95 °C for 3 min, denaturation at 95 °C for 30 s, followed by annealing at 56.5 °C for 30 s and elongation at 72 °C for 1 min with the final step of extension at 72 °C for 5 min. Similarly, amplification of the *agrA* gene was performed for 30 cycles with the same conditions, except that the annealing temperature was set at 56 °C. (Divyakolu et al., 2021). PCR amplicons were resolved by electrophoresis on 1.5% agarose gels stained with ethidium bromide and visualized under UV illumination using a gel documentation system (Bio-Rad, USA). MRSA and MSSA reference strains were used as positive and negative controls, respectively.

**Table 1:** Primer sequences for amplification of target genes.

**Target gene**	**Primer**	**Primer sequence (5^′^3^′^)**	**Product size (bp)**	**Annealing temperature °C**
*mecA*	*mecA* F	5′-GTAGAAATGACTGAACGTCCG-3′	310	56.5 °C
	*mecA* R	5′-CCAATTCCACATTGTTTCGG-3′		
*agrA*	*agrA* F	5′-GCCTATGGAAATTGCCCTC-3′	163	56 °C
	*agrA* R	5′-GCATGACCCAGTTGGTAAC-3′		
16S rRNA	16S rRNA F	5′-TGTCGTGAGATGTTGGG-3′	270	55 °C
	16S rRNA R	5′-CGATTCCAGCTTCATGT-3′		

### 2.8 Assessment of bacterial viability

To confirm the viability of bacterial cells following treatment with BACs at MIC concentrations, a colony-forming unit (CFU) assay was performed. MRSA (ATCC 43300) and MSSA (ATCC 25923) strains were grown overnight and then treated with curcumin and eugenol at their respective MIC concentrations for 24 h at 37 °C. Following incubation, cultures were serially diluted from 10^−1^ to 10^−5^ in sterile PBS. From each dilution, 100 μL was plated on Mueller-Hinton Agar (MHA) plates in triplicate and incubated at 37 °C for 24 hours. Only plates from the 10^−3^ dilution were selected for colony enumeration and comparative visualization, as they consistently yielded distinct, countable colonies across all treatment groups. The presence of visible colonies was interpreted as evidence of bacterial viability after BACs exposure (Rahman and Butzin, 2024).

### 2.9 RNA extraction and cDNA synthesis

The total RNA was extracted from treated and untreated cells using RNAiso Plus reagent (Takara Bio Inc., Japan), following the manufacturer's protocol. For the untreated batch, the strains (VITKV25, VITKV32, VITKV39) and MRSA, MSSA controls were inoculated in Nutrient broth and incubated overnight at 37 °C. For the treated batch, the overnight cultures of the isolates (VITKV25, VITKV32, VITKV39) and MRSA, MSSA controls were treated with curcumin, eugenol, and gallic acid at their respective MIC values for 24 h. The rationale for using MIC concentrations was to capture a transcriptional snapshot under growth-inhibited conditions, allowing us to examine how exposure to these BACs affects the gene expression. Despite inhibition of visible growth, sufficient intact bacterial cells remained for successful RNA extraction and cDNA synthesis. Then both the untreated and treated strains were taken to extract total RNA using RNAiso Plus. Briefly, the bacterial cells were harvested by centrifugation at 10,000 rpm for 10 min. the pellet was resuspended in RNAiso Plus reagent and subjected to mechanical disruption using sterile glass beads with intermittent vortexing to ensure efficient cell lysis. Phase separation was carried out by chloroform addition and centrifugation, followed by RNA precipitation using isopropanol. The RNA pellet was washed with 75% ethanol, air-dried, and resuspended in RNase-free water. RNA concentration and purity were determined using a NanoDrop spectrophotometer (Thermo Fisher Scientific, USA) before cDNA synthesis. First-strand cDNA synthesis was performed using the ABScript II cDNA first-strand synthesis kit (Abclonal, China) according to the manufacturer's protocol. The cDNA samples were stored at −20 °C until further use in qRT-PCR analysis.

### 2.10 Gene expression analysis by qRT-PCR

The qRT-PCR was carried out using Genious 2X SYBR Green Fast qPCR mix (Abclonal, China) following the manufacturer's instructions. The primers used in this study targeted *mecA, agrA* and 16S rRNA (housekeeping gene as an internal control) (Divyakolu et al., 2021). Amplification was performed using a Bio-Rad real-time PCR system at 95 °C for 5 min, by the subsequent steps: 35 cycles of 95 °C for 15 s, 56 °C for 30 s and 60 °C for 1 min. All samples were analyzed in triplicate. Expression levels of *mecA* and *agrA* were normalized to the 16S rRNA housekeeping gene, and relative expression was calculated using the 2^∧^–ΔΔCT technique. MRSA, MSSA reference strains were used as controls. Also, to confirm the absence of genomic DNA contamination, no-reverse-transcriptase (–RT) control reactions were performed, and melt peak analysis showed no specific amplification (see Supplementary Figure S4). Primer efficiencies were determined using five 10-fold serial dilutions of cDNA, and all primer pairs (*mecA, agrA*, 16S rRNA) demonstrated amplification efficiencies within the acceptable range (96.8–98.7%) and high linearity (R^2^ ≥ 0.996), as shown in Supplementary Table S1.

### 2.11 Statistical analysis

All experiments were conducted with two independent biological replicates. The CV assay, MIC, and qRT-PCR were each performed in triplicate. Results are presented as mean ± standard deviation (SD). For all graphical representations, optical density (OD) and percentage values are indicated above the bars. Statistical analysis was performed using one-way analysis of variance (ANOVA) followed by Tukey's multiple comparison test. A *p*-value of <0.001 (*p* < 0.001) was considered statistically significant. All statistical analysis and data visualizations were performed using Origin 2024b software (OriginLab Corporation, USA).

## 3 Results

### 3.1 *S. aureus* prevalence among patients

In this study, a total of 150 nasal swab samples were equally collected from male (*n* = 75) and female (*n* = 75) patients. Out of these, 53 isolates (35.3%) were identified as *S. aureus* isolates based on morphological and biochemical characterization. The proportion of *S. aureus* positive isolates was higher among females (45.3%) compared to males (25.3%), and this difference was statistically significant (χ^2^ = 6.56, *p* = 0.01). On MSA, the presumptive isolates formed yellow-colored colonies with surrounding yellow zones. Gram staining revealed Gram-positive cocci in clusters. The isolates also tested positive for catalase (bubble formation), tube coagulase (visible clot formation), and DNase (zone of clearance on DNase agar). The oxidase test was negative, as indicated by no color change on the oxidase disc. A summary of the phenotypic test results is presented in Table 2.

**Table 2:** Morphological and biochemical characterization of presumptive *S. aureus* isolates (*n* = 150).

**S. No**.	**Tests**	**Positive (*n*, %)**	**Negative (*n*, %)**	**Presumptive *S. aureus* profile**
1	Gram staining	**96 (64%)**	54 (36%)	*n* = 53, 35%
2	Mannitol fermentation	**71 (47%)**	79 (53%)	
3	Coagulase	**63 (42%)**	87 (58%)	
4	Catalase	**65 (43%)**	85 (57%)	
5	Oxidase	92 (61%)	**58 (39%)**	
6	DNase	**53 (35%)**	97 (65%)	

The bold values represent the positive outcome for each biochemical test.

### 3.2 Antibiotic susceptibility profiling of *S. aureus* isolates

**Figure 1:**
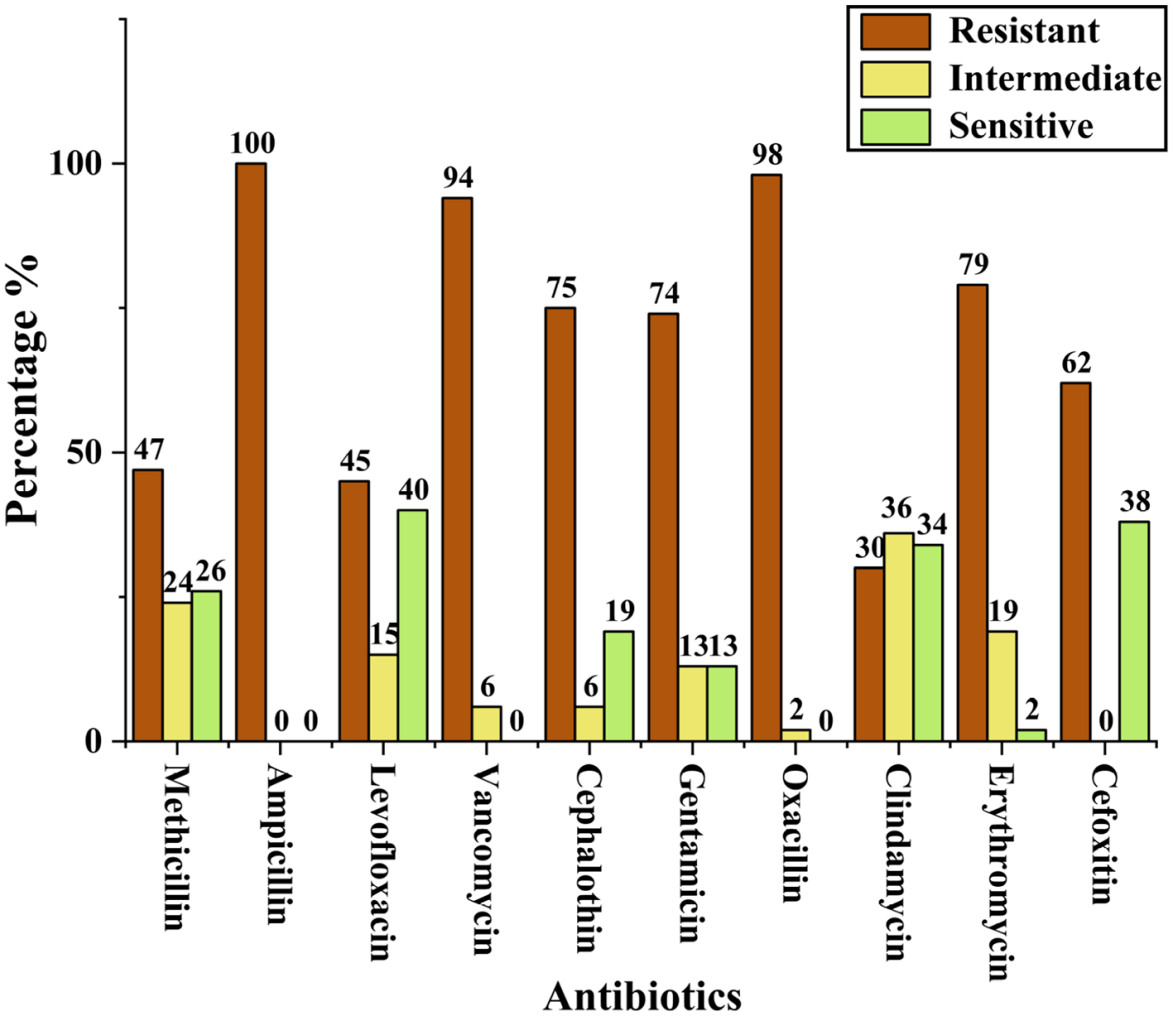
Bar graph representing the percentage of presumptive *S. aureus* isolates categorized as resistant, intermediate, or susceptible to each antibiotic tested by the antibiotic disc diffusion method.

**Figure 2:**
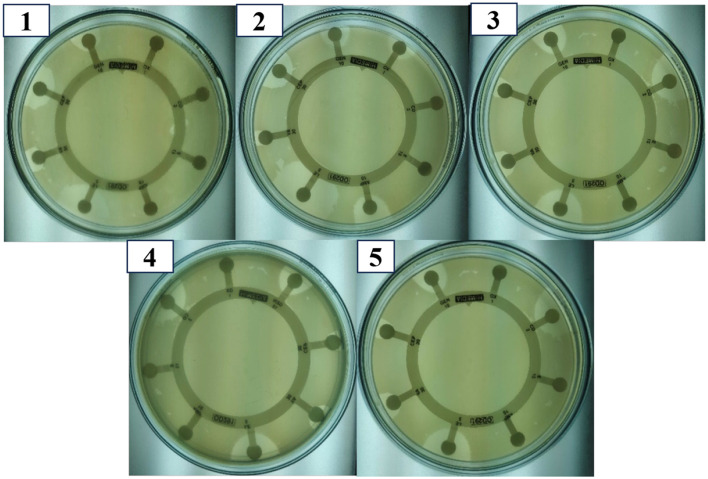
Antibiotic sensitivity pattern of presumptive *S. aureus* isolates using Octo disc in MHA by the antibiotic disc diffusion method. The clear inhibition zones around antibiotic discs indicate varying degrees of susceptibility. Images shown are representative and do not correspond to a specific strain.

Antibiotic susceptibility testing was performed for all 53 presumptively confirmed *S. aureus* isolates using the disc diffusion method. The isolates exhibited a high level of resistance to multiple antibiotics. Resistance to ampicillin (100%) was most significant, followed by oxacillin (98%) and vancomycin (94%). In contrast, lower resistance was observed against clindamycin (30%). Methicillin resistance was assessed using cefoxitin disc diffusion, which identified 62% (25/53) of the isolates as MRSA. Figure 1 presents the resistance profile of all isolates based on the disc diffusion assay, showing the percentage of resistance, intermediate susceptibility, and sensitivity across the tested antibiotics. Representative disc diffusion results are shown in Figure 2, the distribution of resistance levels among the isolates is summarized in Table 3, and the three isolates (VITKV25, VITKV32, and VITKV39) that demonstrated resistance to all 10 antibiotics tested were highlighted. These multidrug-resistant strains were selected for further analysis involving compound treatment and gene expression profiling.

**Table 3:** Antibiotic resistance distribution among clinical *S. aureus* isolates (*n* = 53).

**Resistance profile (no. of antibiotics)**	**No. of isolates**	**Percentage (%)**
1	0	0%
2	1	2%
3	0	0%
4	3	5%
5	8	15%
6	6	11%
7	10	19%
8	13	24%
9	9	17%
**10**	**3**	**6%**
>3	52	98%

Values in bold represent isolates resistant to the highest number of antibiotics (10 in total).

### 3.3 Molecular identification of *S. aureus* isolates through 16S rRNA sequencing

**Figure 3:**
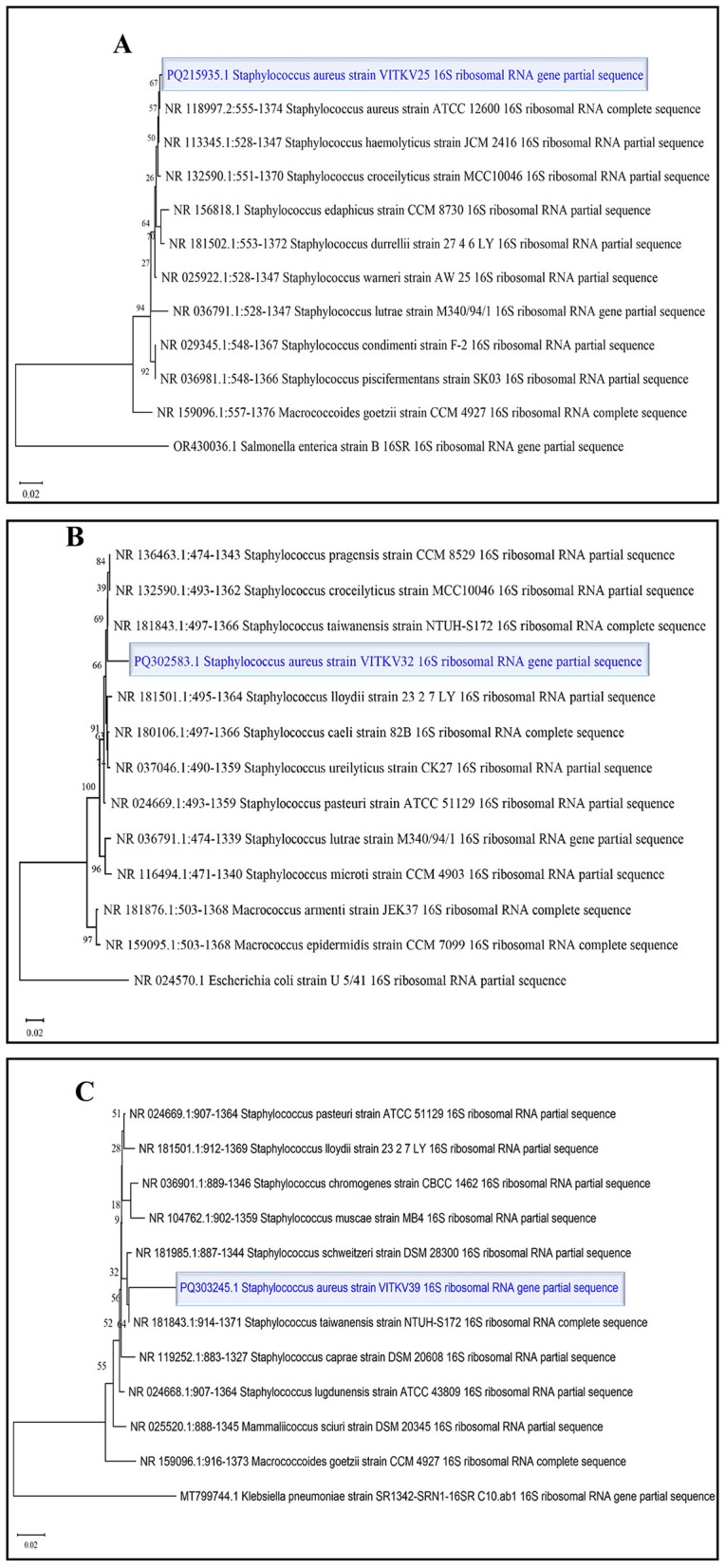
Phylogenetic tree analysis of *S. aureus* isolates. Neighbor-joining phylogenetic trees were constructed for clinical isolates **(A)** VITKV25, **(B)** VITKV32, and **(C)** VITKV39 using partial 16S rRNA gene sequences—each isolate (highlighted) clustered closely with *S. aureus* reference strains from GenBank, confirming species-level identity.

Three multidrug-resistant isolates (VITKV25, VITKV32, and VITKV39), which demonstrated resistance to all 10 antibiotics tested were selected for molecular identification. The isolates were subjected to 16S RNA gene sequencing, and the results sequences were analyzed using the NCBI BLASTn tool. All three isolates showed high sequence similarity to *S. aureus* strains in the GenBank database. The isolates were confirmed as *S. aureus* and the corresponding nucleotide sequences were submitted to GenBank with accession numbers PQ215935 (VITKV25), PQ302583 (VITKV32), and PQ303245 (VITKV39). A phylogenetic tree was constructed using the neighbor-joining method in MEGA11 to assess the evolutionary relationships of the sequenced isolates with reference Staphylococcus species. As shown in Figure 3, all three isolates clustered closely with known *S. aureus* sequences, further supporting their taxonomic classification.

### 3.4 Biofilm formation assay

**Figure 4:**
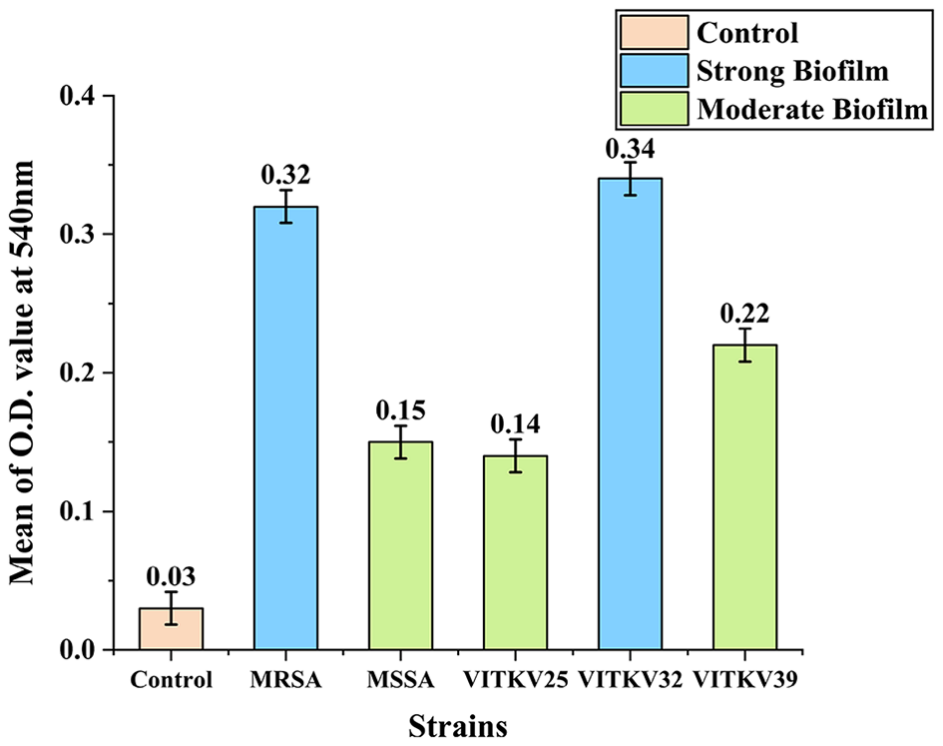
Biofilm formation in clinical and reference *S. aureus* strains. Bar chart representing the mean absorbance values at 540 nm (±SEM) from the crystal violet assay for clinical isolates VITKV25, VITKV32, and VITKV39 and control strains (*S. aureus* ATCC4300 and ATCC 25923). The TSB-only control showed negligible biofilm formation. VITKV32 and MRSA control strain were categorized as strong biofilm producers, while VITKV25, VITKV39, and MSSA control strain demonstrated moderate biofilm formation. Data represent the mean of three independent replicates.

The crystal violet assay results confirmed that all three multidrug-resistant *S. aureus* isolates were biofilm producers. Absorbance values at 540 nm were used to classify biofilm strength. Isolate VITKV32 exhibited strong biofilm formation with a mean absorbance of 0.34, while VITKV25 (0.14) and VITKV39 (0.22) were categorized as moderate biofilm producers. The positive control strains MRSA and MSSA reference strains, showed strong (0.32) and moderate (0.15) biofilm formation, respectively. The negative control (TSB only) exhibited negligible absorbance (0.03), confirming assay specificity. As shown in Figure 4, biofilm formation among the clinical isolates was comparable to that of standard control strains. The results are expressed as the mean of three biological replicates, with error bars representing standard error of the mean (SEM).

### 3.5 Antibacterial activity of BACs

**Figure 5:**
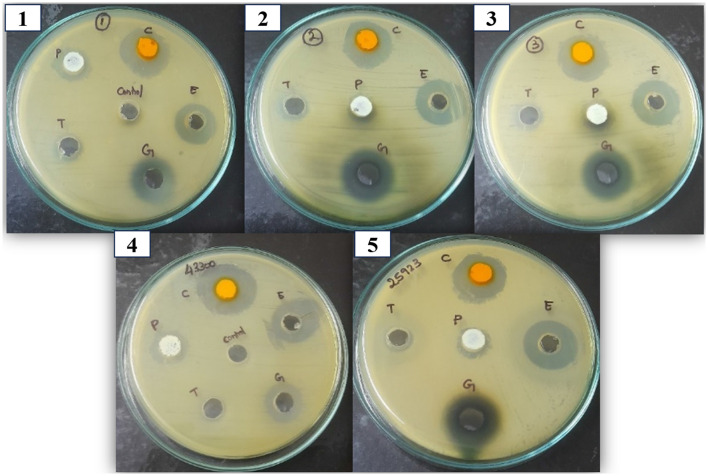
Antibacterial activity of the BACs against clinical and control *S. aureus* isolates. The zone of inhibition denotes the antibacterial activity of compounds against the *S. aureus* strains. 1,2, and 3 correspond to VITKV25, VITKV32, and VITKV39, respectively. 4 and 5: MRSA and MSSA reference strains respectively. C, curcumin; E, Eugenol; G, Gallic acid; T, α-terpineol; P, Piperine. DMSO was used as the negative control.

The selected BACs—curcumin, eugenol, piperine, gallic acid, and α-terpineol—were chosen to represent distinct classes of bioactive secondary metabolites, including polyphenols (gallic acid), diarylheptanoids (curcumin), phenolic monoterpenes (eugenol), alkaloids (piperine), and monoterpenoids (α-terpineol). This chemical diversity was intentional, allowing us to evaluate the broad-spectrum potential of structurally different phytocompounds against *S. aureus*. These BACs were evaluated against the multidrug-resistant *S. aureus* isolates using the agar well diffusion method. The inhibitory effect was evaluated based on the diameter of the zone of inhibition. Among the tested compounds, curcumin, eugenol, and gallic acid exhibited measurable zones of inhibition against all three isolates, indicating effective antibacterial activity. Meanwhile, piperine and α-terpineol did not produce any visible zones at the tested concentrations and were considered inactive under these conditions. The results were summarized in Figure 5, which denotes the zone of inhibition produced by each compound against the clinical isolates. Based on these findings, curcumin, eugenol, and gallic acid were selected for further evaluation through MIC assays.

### 3.6 MIC determination of BACs

**Figure 6:**
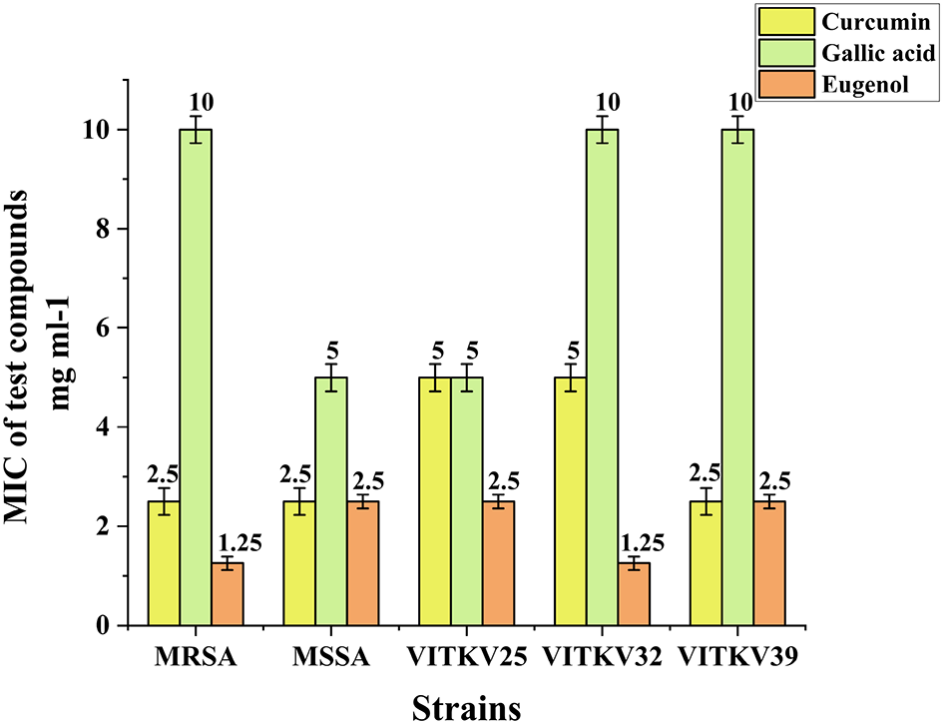
Bar graph representing the MIC values (mg/mL) of curcumin, eugenol, and gallic acid against reference strains (MRSA & MSSA) clinical *S. aureus* isolates (VITKV25, VITKV32, VITKV39). Data represent mean ± standard error of triplicate experiments.

Based on the results of the agar well diffusion assay, the MICs of curcumin, eugenol, and gallic acid were determined by the broth microdilution method. All three compounds exhibited inhibitory effects against the isolates and reference strains, with compound-specific and strain-specific variability. Curcumin showed the highest potency with MIC values of 2.5 mg/mL against MRSA, MSSA, VITKV25, and VITKV39 and the lowest MIC of 1.25 mg/mL against VITKV32. Eugenol also demonstrated strong activity with MICs of 1.25 mg/mL for MRSA and VITKV32 and 2.5 mg/mL for all other strains. In contrast, gallic acid exhibited comparatively lower efficacy, with MICs of 10 mg/mL for MRSA, VITKV32, and VITKV39 and 5 mg/mL for MSSA and VITKV25. The comparative MIC values are graphically represented in Figure 6. These findings indicate that curcumin and eugenol are more effective than gallic acid in inhibiting the growth of multidrug-resistant *S. aureus* strains.

### 3.7 Molecular detection of *mecA* and *agrA* genes by PCR

**Figure 7:**
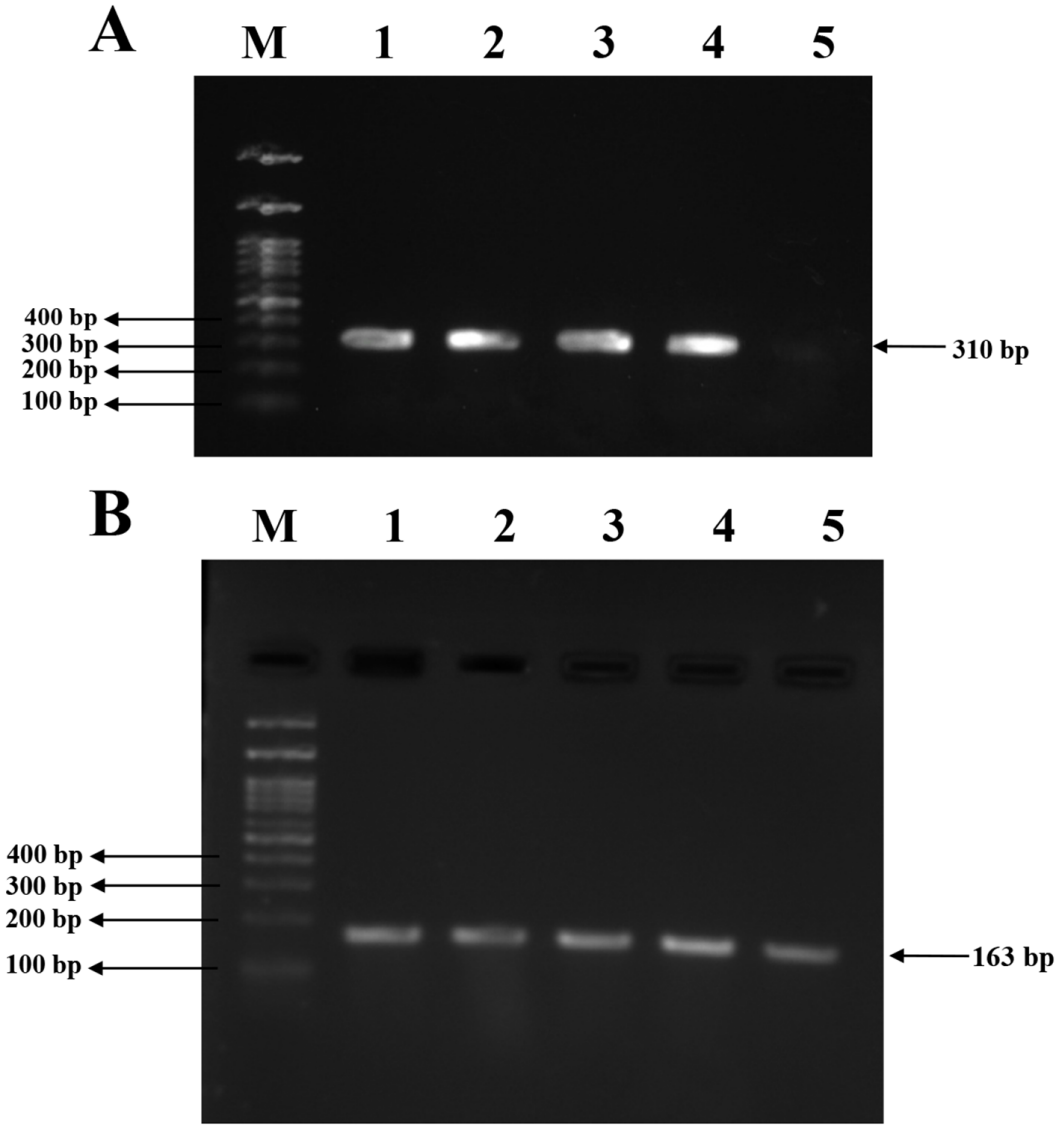
PCR amplification for *mecA* and *agrA* genes in clinical and reference *S. aureus* strains. **(A)** Amplification of the *mecA* gene (310bp) in *S. aureus* isolates. Lane 1, 2, 3: clinical *S. aureus* isolates VITKV25, VITKV32, VITKV39, respectively; Lane 4: positive control (MRSA control strain); Lane 5: negative control (MSSA control strain); Lane M: 100 bp DNA ladder. **(B)** Amplification of the *agrA* gene (163 bp) across isolates. Lane 1,2,3: VITKV25, VITKV32, and VITKV39; Lanes 4 and 5: MRSA and MSSA control strains, respectively; Lane M: 100 bp DNA ladder. All isolates tested positive for *agrA*, while only MRSA strains showed *mecA* amplification.

Conventional PCR was performed to detect the presence of the *mecA* and *agrA* genes in the isolates. Amplification of the *mecA* gene yielded a product of 310bp, which was observed in all three clinical isolates and the MRSA reference strain. No amplification was detected in the MSSA control, confirming its *mecA*-negative phenotype. Subsequently, amplification of the *agrA* gene resulted in a 163bp product in all tested strains, which includes three clinical isolates, MRSA and MSSA controls, indicating the presence of a functional agr quorum sensing system across both resistant and susceptible strains. The PCR amplicons were visualized using 1.5% agarose gel electrophoresis, and the results are illustrated in Figure 7.

### 3.8 Viability assessment

The bacterial cells remained viable following exposure to curcumin and eugenol at MIC levels, a CFU assay was conducted on MRSA and MSSA strains. Cultures were serially diluted and plated after 24 h of treatment. As shown in Supplementary Figure S1, the 10^−3^ dilution consistently produced visible, countable colonies in both treated and untreated groups. The presence of colonies in curcumin- and eugenol-treated cultures confirmed that growth was inhibited but not eliminated, supporting the feasibility of RNA extraction and downstream qRT-PCR analysis.

### 3.9 Gene expression analysis of *mecA* and *agrA* by qRT-PCR

**Figure 8:**
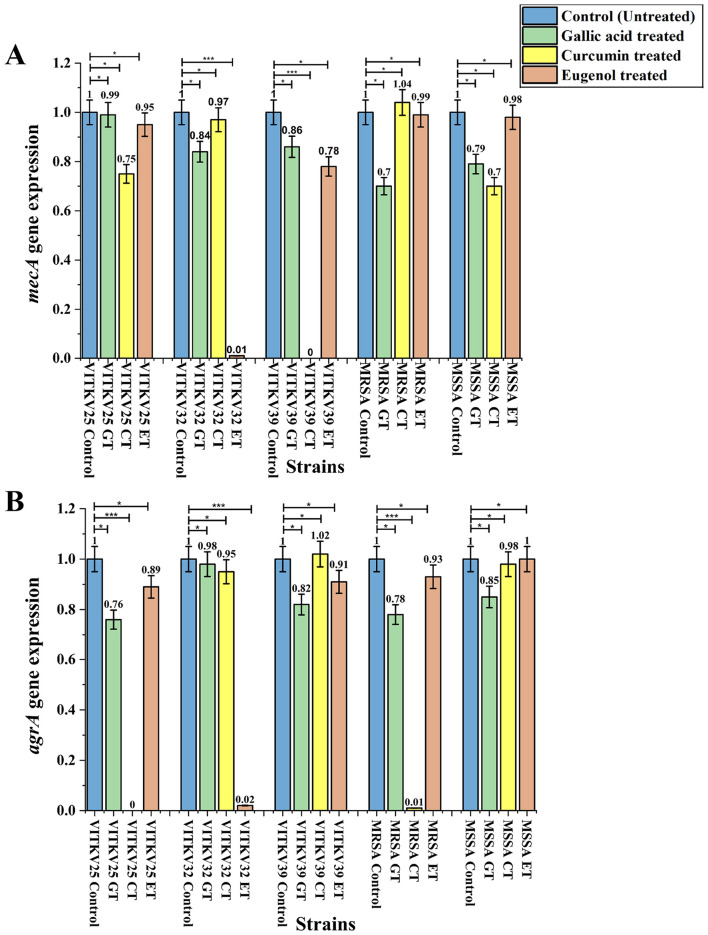
Relative gene expression analysis of *mecA* **(A)** and *agrA* **(B)** gene expression in *S. aureus* isolates treated with gallic acid (GT), curcumin (CT), and eugenol (ET). Expression levels were normalized to 16S rRNA and are represented relative to untreated controls (set as 1.0). Significant downregulation of *mecA* was observed in VITKV39 (CT) and VITKV32 (ET), while *agrA* gene expression was effectively decreased in VITKV25 (CT), MRSA (CT), and VITKV32 (ET). Data represent the mean ± SEM from three independent experiments. ***p < 0.001, *p < 0.05.

**Figure 9:**
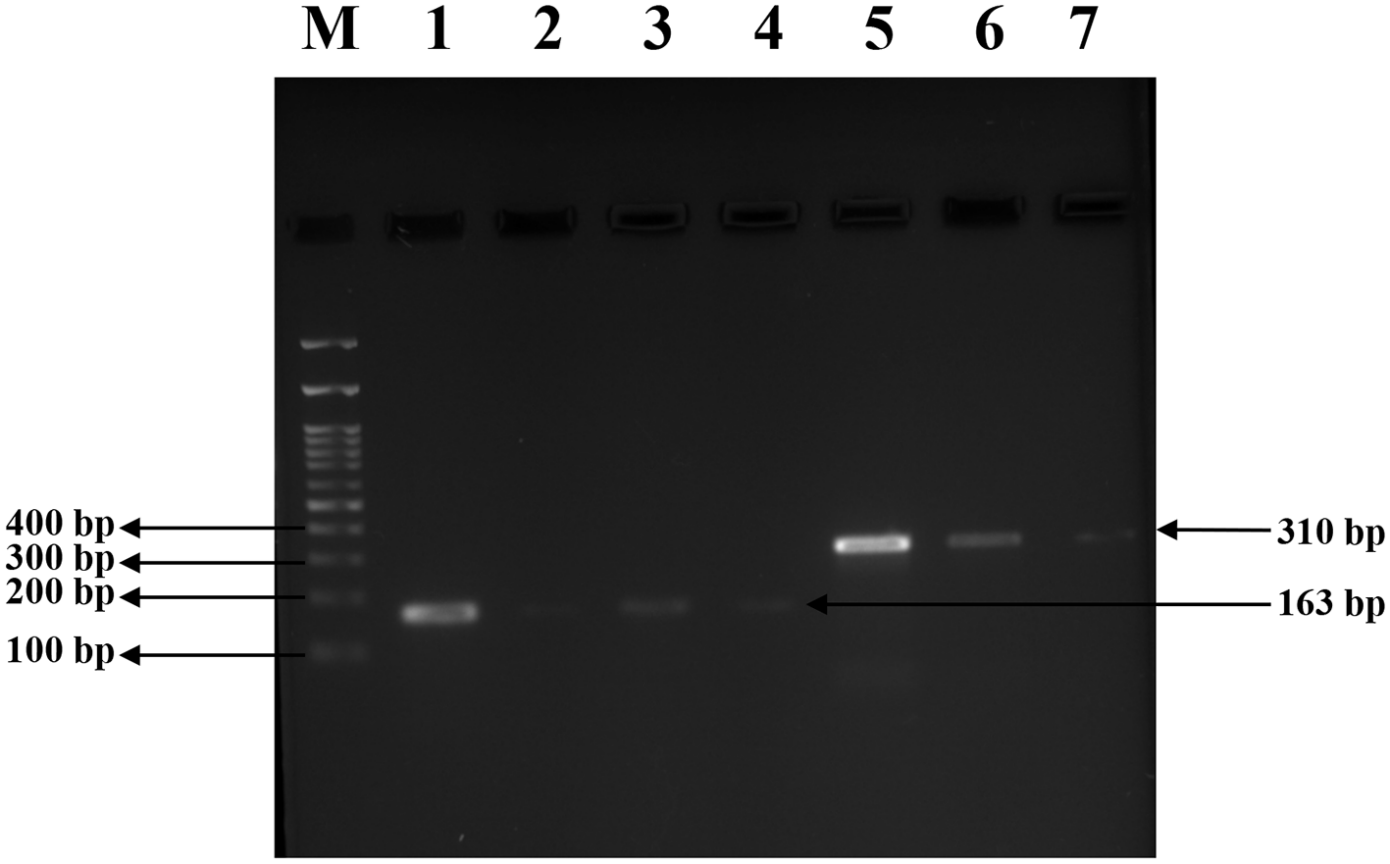
Agarose gel electrophoresis for visualizing the downregulated gene expression following BACs treatment. Gel image indicating PCR products of *mecA* (310 bp) and *agrA* (163 bp) amplified from treated *S. aureus* strains. Lane M: 100 bp DNA ladder; Lane 1: positive control for *agrA* (ATCC43300); Lanes 2, 3 and 4: *agrA* amplification from VITKV25 (CT), and MRSA (CT), and VITKV32 (ET), respectively; Lane 5: positive control for *mecA* (ATCC 43300); Lane 6,7: *mecA* amplification from VITKV39 (CT) and VITKV32 (ET), respectively. Reduced band intensity in treated samples indicates transcriptional suppression of target genes.

The relative expression levels of the *mecA* and *agrA* genes in the multidrug-resistant *S. aureus* isolates after treatment with BACs were evaluated using qRT-PCR. The expression levels were normalized to the 16S rRNA housekeeping gene, and it was assessed by comparing treated cultures to untreated controls using the 2^∧^–ΔΔCt method. Both *mecA* and *agrA* gene expressions varied across the treated *S. aureus* isolates. Curcumin treatment significantly downregulated both genes in multiple strains (*p* < 0.001). Notably, *mecA* expression in VITKV39 was completely suppressed (0.00 ± 0.00), and *agrA* expression in VITKV25 and MRSA was also strongly reduced (0.00 ± 0.01, respectively). Eugenol produced a moderate but statistically significant decrease in gene expression, particularly in VITKV32 (*mecA*: 0.01 ± 0.00; *agrA*: 0.02 ± 0.01; *p* < 0.001). In contrast, gallic acid had no significant effect on *mecA* gene expression and only a modest effect on *agrA* expression (*p* < 0.05). The expression values were normalized to untreated controls, which were set at 1.0. The results are represented as mean ± SEM from three individual experiments. The Supplementary Figure S5 demonstrates the amplification of the reference gene 16S rRNA by PCR. The fold changes in *mecA* and *agrA* expression following compound treatment are summarized in Figure 8. Furthermore, PCR analysis was performed to study the downregulated strains, and the results are presented in Figure 9. In addition to transcriptional modulation, an antibiotic synergy assay was performed to evaluate whether curcumin and eugenol enhance the efficacy of β-lactam antibiotics against MRSA. Combination treatments using sub-inhibitory concentrations (½ MIC) of BACs with methicillin and ampicillin were assessed via agar well diffusion. As shown in Supplementary Figure S2, the combination treatments resulted in larger zones of inhibition compared to antibiotics alone, suggesting a potential synergistic or additive effect of BACs in re-sensitizing MRSA to β-lactam antibiotics. The effect of BACs on MRSA biofilm formation was assessed using the crystal violet assay. Treatment with curcumin, and eugenol resulted in a marked reduction in biofilm formation across all three clinical MRSA isolates. Based on OD540 values, treated strains showed a shift from strong to moderate or non-biofilm producers, suggesting a functional impact on biofilm development. These findings are presented in Supplementary Figure S3.

## 4 Discussion

MRSA infections have emerged as a major pathogen in both the general public and clinical settings, contributing significantly to antimicrobial resistance challenges. MRSA isolates typically carry the *mecA* gene, which encodes the PBP2a and confers resistance to β-lactam antibiotics, particularly cefoxitin and penicillin. However, some MRSA strains may remain sensitive to other classes of antibiotics depending on the local resistance ecology and clinical exposure history (Idrees et al., 2023). In the present study, the prevalence of MRSA among nasal *S. aureus* isolates was 16% (25 out of 150), which aligns with previous reports of nasal MRSA carriage in healthcare environments in India, where rates range from 12% to 20% (Wolde et al., 2023). The prevalence of *S. aureus* was higher in female subjects, with an overall carriage rate of 35.3%, and these results highlight the importance of routine screening and decontamination techniques, particularly in hospital settings, to prevent infection and control the spread of MRSA (Aravind et al., 2000).

The phenotypic and biochemical assays, including cefoxitin resistance, catalase, coagulase, DNase, and mannitol fermentation tests, were employed to identify *S. aureus* strains. Initial screening and identification of *S. aureus* isolates in this study were performed using a combination of standard phenotypic assays. Gram-staining facilitated the preliminary classification of Gram-positive cocci. Subsequent growth on MSA, characterized by yellow colonies due to mannitol fermentation in high-salt conditions, served as a selective and differential indicator for presumptive *S. aureus* (Thakur et al., 2017). Further, these observations were supported by positive results in the coagulase test, which remains a gold standard for differentiating *S. aureus* from other coagulase-negative *Staphylococcus* species (Sperber and Tatini, 1975). The catalase test helped differentiate *Staphylococci* spp. from catalase-negative *Streptococci* spp. (Mustafa, 2014). The oxidase test, which detects the presence of cytochrome *c* oxidase, was negative in all presumptive *S. aureus* isolates, consistent with the known oxidase-negative profile of *Staphylococcal* species and helping to exclude oxidase-positive genera such as *Neisseria* and *Pseudomonas* (Arjyal et al., 2020). The DNase test determines whether an organism can produce the DNase enzyme, which degrades DNA into smaller fragments that the cell can absorb. This test can distinguish *S. aureus* from other *Staphylococcus* species. Gram-positive, catalase-positive cocci that produce DNase can potentially be identified as *S. aureus* (Kateete et al., 2010). Altogether, these assays provided a comprehensive and reliable phenotypic profile. Out of 150 total isolates, 53 were confirmed as presumptive *S. aureus* using this phenotypic strategy, establishing the basis for downstream antimicrobial resistance and molecular characterizations.

The antibiotic susceptibility testing using the Kirby-Bauer disc diffusion method remains a standard phenotypic approach for evaluating bacterial resistance. In this study, cefoxitin, methicillin, and oxacillin were among the β-lactam agents used to determine resistance profiles, with interpretive breakpoints referenced from CLSI guidelines (Jorgensen and Ferraro, 2009). The zone of inhibition diameters provided a reliable estimate of antibiotic efficacy, and the consistent resistance observed to cefoxitin helped to confirm MRSA phenotypes among the selected isolates. Three isolates (VITKV25, VITKV32, and VITKV39) exhibited complete resistance to all ten antibiotics tested. These extensively drug-resistant profiles prompted their selection for molecular identification and further analysis. The molecular characterization by 16S rRNA sequencing results confirmed all three strains as *S. aureus*, with high sequence similarity to reference strains in GenBank, as validated by phylogenetic tree analysis.

The QS system in *S. aureus*, predominantly mediated by the agr operon, coordinates population-dependent gene regulation related to virulence, adhesion, motility, and notable biofilm formation. In MRSA, biofilm development plays a crucial role in persistent infections by enhancing bacterial survival on host tissues and medical devices, impeding phagocytosis, and restricting antibiotic penetration (Yamazaki et al., 2024). In this study, the crystal violet biofilm assay revealed that VITKV32 was a strong biofilm producer, while VITKV25 and VITKV39 exhibited moderate biofilm-forming capabilities, relative to MRSA and MSSA reference strains. These findings are consistent with recent reports, including those by B. Chaudhary et al., which indicate that biofilm production among clinical MRSA isolates varies widely, with stronger biofilm phenotypes frequently associated with co-expression of resistance determinants and virulence factors (Chaudhary et al., 2021). These insights reinforce the clinical significance of assessing biofilm potential alongside resistance profiling, particularly in multidrug-resistant strains, where biofilm formation increases the risk of chronic infection in addition to antimicrobial resistance.

The antibacterial activity of selected compounds was first evaluated through agar well diffusion, which allowed for a preliminary assessment of efficacy against the clinical isolates. Compounds were chosen based on their natural sources and recent research highlighting their antibacterial effects against *S. aureus*. Among all five tested compounds, curcumin, eugenol, and gallic acid produced clear inhibition zones and were selected for further analysis. In contrast, piperine and α-terpineol showed no measurable activity under the tested conditions. MIC assays were performed to determine the potency of these compounds. The MIC values revealed that curcumin exhibited the highest inhibitory activity, followed by eugenol, while gallic acid showed relatively lower activity. The outcomes are consistent with previous studies, where curcumin demonstrated strong antibacterial activity against *S. aureus* isolates as reported by Teow et al. (2016), the notable efficacy of eugenol is demonstrated by Li et al. (2024) and gallic acid exhibited moderate activity in line with observations by Jiamboonsri et al. (2023). These results, identifying curcumin and eugenol as the most potent inhibitors, provided a rational basis for selecting these compounds to investigate their effects on gene expression at their respective MICs.

Followed by the MIC profiling, the PCR amplification confirmed the presence of the *mecA* and *agrA* genes in all three isolates, with MRSA and MSSA reference strains used as positive and negative controls, respectively. Methicillin resistance was molecularly confirmed by the identification of *mecA*, which was consistent with the previously noted phenotypic cefoxitin resistance. The detection of *mecA* justify the classification of these strains as MRSA as it is dependable diagnostic marker in clinical microbiology (Dhungel et al., 2021). A functional QS system that supports the regulatory control of virulence was confirmed by the presence of *agrA*. Also, its structural susceptibility to small-molecule inhibition, *agrA* is increasingly recognized as a promising ant virulence target (Podkowik et al., 2023). These findings are reinforced by recent clinical surveillance data in which *mecA* was found in 95% of MRSA strains and *agrA* in 85% of the same isolates, according to a study that examined *S. aureus* from hospital settings (Eidaroos et al., 2025). Therefore, these molecular confirmations established the basis for evaluating the transcriptional modulation of virulence and resistance genes in response to BACs treatment.

The expression of *mecA* and *agrA* relative to the 16S rRNA housekeeping gene was evaluated by qRT-PCR to investigate the transcriptional effects of BACs treatment on the clinical isolates. Remarkably, *mecA* gene expression was downregulated in curcumin-treated VITKV39 and eugenol-treated VITKV32, whereas *agrA* was suppressed in curcumin-treated VITKV25 and the MRSA reference strain, as well as in eugenol-treated VITKV32. These findings depicted that both curcumin and eugenol have gene-specific inhibitory effects on significant resistance and virulence factors in *S. aureus*. Systematically, the transcriptional suppression of the *mecA* and *agrA* genes may involve interference with upstream regulatory elements. Our study results are consistent with the previous studies that have demonstrated curcumin's ability to downregulate *mecA* gene expression, leading to a decrease in PBP2a levels and restoring the efficacy of β-lactam antibiotics against MRSA strains (Teow et al., 2016). Additionally, curcumin has been reported to inhibit the *agrA* gene, impacting the QS system, attenuating virulence, and biofilm formation (Kashi et al., 2024). Curcumin's antimicrobial activity has also been attributed to its ability to compromise bacterial cell wall integrity. Previous studies have shown that curcumin increases membrane permeability, alters lipid composition, and causes structural disintegration of the cell wall, ultimately leading to cytoplasmic leakage and cell death (Mun et al., 2014). Such membrane damage may activate stress-responsive regulatory pathways in *S. aureus*, which could contribute to the observed downregulation of *mecA* and *agrA* in our study. Eugenol has also demonstrated significant anti-biofilm activity and QS disruption in *S. aureus*, which may indirectly affect *mecA* expression and enhance susceptibility to antibiotics (El-Far et al., 2021). Also, eugenol is known to disrupt membrane integrity and may alter signal transduction through sensor kinase like AgrC, indirectly suppressing *AgrA* activation. Although this study focused on *mecA* and *agrA* as key targets, ongoing work includes the transcriptional analysis of additional regulators and virulence genes such as *SarA, blaZ, icaA*, and *hla*. The role of *diadenylate cyclase* in biofilm regulation is also acknowledged and will be explored in future transcriptomic studies.

Antimicrobial-induced transcriptional changes are often attributed to indirect effects arising from cellular stress, rather than direct modulation of gene expression. As outlined by Kashi et al. (2024), compounds may alter transcriptional profiles through disruption of membrane integrity or metabolic processes without specifically targeting transcription factors. In this context, the observed downregulation of *mecA* and *agrA* in response to BACs may reflect secondary stress responses. Further mechanistic studies are required to determine whether these effects result from direct regulatory interference (Kashi et al., 2024).

## 5 Conclusion

This study confirms the prevalence of MRSA and multidrug-resistant *S. aureus* in nasal carriage within a clinical environment, with a prevalence rate of 16% (25/150). Among the BACs tested, curcumin and eugenol exhibited significant antibacterial activity and also downregulated key resistance and virulence genes at their MIC concentrations. Curcumin effectively inhibited *mecA* in VITKV39 and *agrA* in both VITKV25 and MRSA, while eugenol inhibited *mecA* and *agrA* in VITKV32. While gene expression modulation may be influenced in part by growth inhibition, the consistent transcriptional downregulation observed suggests an additional regulatory effect of these compounds. The study outcomes underscore the potential of curcumin and eugenol as promising phytocompounds by targeting both the *mecA* gene for antimicrobial resistance and the *agrA* gene for QS-mediated virulence pathways in MRSA. Such mechanisms could help reduce pathogenicity and may enhance the efficacy of β-lactam antibiotics against resistant strains. This dual-action approach can help limit the risk of chronic infection and the development of multidrug-resistant strains. Future studies exploring quorum quenching, anti-virulence mechanisms, and synergy with antibiotics are crucial to advance these compounds toward clinical application.

## Data Availability

The original contributions presented in the study are included in the article/Supplementary material, further inquiries can be directed to the corresponding author.
