# Supplementary material for: Targeted inhibition of mecA and agrA genes in clinical MRSA isolates by natural bioactive compounds
Source: Front Microbiol. 2025 Aug 26;16:1643774. doi: 10.3389/fmicb.2025.1643774 (PMC12417121; doi:10.3389/fmicb.2025.1643774)
Supplement: Supplementary file 1 [file Data_Sheet_1.docx]

**Supplementary file:**

**Supplementary Figure S1** Assessment of bacterial viability. Bacterial cultures were treated with curcumin and eugenol at MIC concentrations for 24 hours and serially diluted (10¹–10⁻⁵). Only plates from the 10⁻³ dilution were used for viability comparison. (A) Untreated MRSA, (B) Untreated MSSA, (C) MRSA + curcumin, (D) MSSA + curcumin, (E) MRSA + eugenol, (F) MSSA + eugenol. Colony growth observed in treated plates confirms the presence of viable cells suitable for RNA extraction.

**Supplementary Figure S2** Agar well diffusion assay**.** This figure illustrates the impact of combining curcumin and eugenol with β-lactam antibiotics on the growth inhibition of MRSA. The experiment was conducted using the agar well diffusion method, where BACs were applied at sub-inhibitory concentrations (½ MIC) in combination with standard concentrations of methicillin or ampicillin. Plate A: 1. Curcumin + Methicillin, 2. Methicillin, 3. Negative control (DMSO), 4. Eugenol + Methicillin. Plate B: 1. Curcumin + Ampicillin, 2. Ampicillin, 3. Negative control (DMSO), 4. Eugenol + Ampicillin. Zones of inhibition were measured after 24 hours of incubation at 37 °C. The visibly enlarged zones in the combination wells (1 and 4 on both plates) compared to antibiotic-only wells (2) suggest that a synergistic or sensitizing effect of BACs may enhance MRSA susceptibility to β-lactam antibiotics.

**Supplementary Figure S3** Biofilm biomass of three clinical MRSA isolates (VITKV25, VITKV32, VITKV39) along with MRSA and MSSA control strains, following treatment with BACs — curcumin (CT), and eugenol (ET) — was quantified using the crystal violet assay. Untreated controls exhibited strong biofilm production (OD_540_ > 0.240), while treated samples showed a significant reduction in biofilm biomass, with some isolates shifting to moderate (0.120–0.240) or non-biofilm-producing levels (OD_540_ < 0.120). Data are presented as mean ± SD from three independent experiments, *p < 0.05. These results indicate the antibiofilm potential of the tested phytocompounds.

**
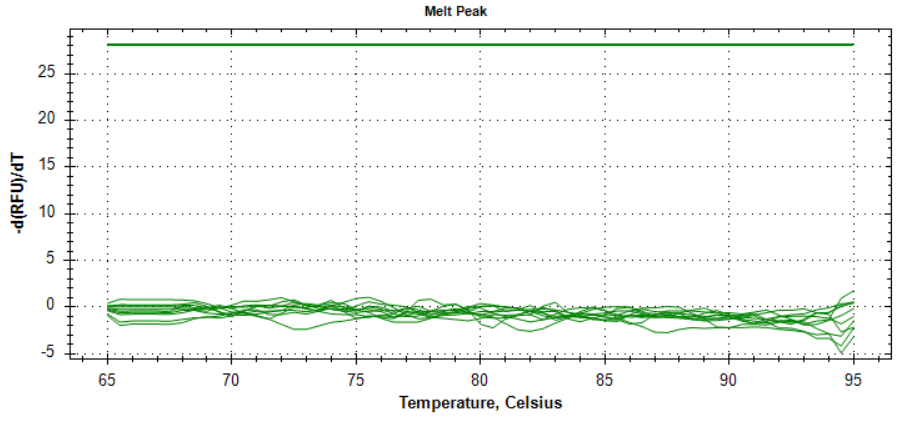
**

**Supplementary Figure 4** Melt peak analysis of no-reverse-transcriptase (–RT) control qPCR reactions. The absence of specific amplification confirms that RNA samples were free of genomic DNA contamination.

**Supplementary Table S1 Primer details and amplification efficiencies used in qRT-PCR analysis**

Amplification efficiency of primer pairs used for qRT-PCR analysis. Efficiency was calculated using standard curves generated from serial 10-fold dilutions of cDNA templates. All primer pairs exhibited high linearity and efficiency, making them suitable for relative quantification using the ΔΔCt method.

| Target gene | Primer pair | Amplicon size (bp) | Annealing Temp (°C) | Slope | R^2^ value | Efficiency (%) |
| --- | --- | --- | --- | --- | --- | --- |
| *mecA* | F- 5'-GTAGAAATGACTGAACGTCCG-3´ | 310 | 56.5 | -3.38 | 0.998 | 97.4 |
|  | R- 5´-CCAATTCCACATTGTTTCGG-3´ |  |  |  |  |  |
| *agrA* | F- 5´-GCCTATGGAAATTGCCCTC-3´ | 163 | 56 | -3.35 | 0.996 | 98.7 |
|  | R- 5´-GCATGACCCAGTTGGTAAC-3´ |  |  |  |  |  |
| 16S rRNA | F- 5´-TGTCGTGAGATGTTGGG-3´ | 270 | 55 | -3.40 | 0.997 | 96.8 |
|  | R- 5´-CGATTCCAGCTTCATGT-3´ |  |  |  |  |  |

**Supplementary Figure 5** PCR for detection of 16S rRNA gene among the *S. aureus* isolates. Agarose gel electrophoresis showing amplification of the 16S rRNA gene (270 bp). Lanes 1 - 5 correspond to VITKV25, VITKV32, VITKV39, MRSA & MSSA, respectively. Lane M: 100 bp DNA ladder. All isolates tested showed positive for amplification, validating RNA integrity and normalization of qRT-PCR.
